# Effects of diazoxide in experimental acute necrotizing pancreatitis

**DOI:** 10.6061/clinics/2017(02)10

**Published:** 2017-02

**Authors:** Roberta de Oliveira Andrade, Tiago Kunitake, Marcia Kiyomi Koike, Marcel C C Machado, Heraldo Possolo Souza

**Affiliations:** Faculdade de Medicina da Universidade de São Paulo, Departamento de Emergências Clínicas, São Paulo/SP, Brazil

**Keywords:** Pancreatitis, Diazoxide, Rats

## Abstract

**OBJECTIVE:**

We aimed to assess the effects of diazoxide on the mortality, pancreatic injury, and inflammatory response in an experimental model of acute pancreatitis.

**METHODS:**

Male Wistar rats (200–400 g) were divided randomly into two groups. Fifteen minutes before surgery, animals received physiological (0.9%) saline (3 mL/kg) (control group) or 45 mg/kg diazoxide (treatment group) *via* the intravenous route. Acute pancreatitis was induced by injection of 2.5% sodium taurocholate via the biliopancreatic duct. Mortality (n=38) was observed for 72 h and analyzed by the Mantel–Cox Log-rank test. To study pancreatic lesions and systemic inflammation, rats (10 from each group) were killed 3 h after acute pancreatitis induction; ascites volume was measured and blood as well as pancreases were collected. Pancreatic injury was assessed according to Schmidt’s scale. Cytokine expression in plasma was evaluated by the multiplex method.

**RESULTS:**

Mortality at 72 h was 33% in the control group and 60% in the treatment group (*p*=0.07). Ascites volumes and plasma levels of cytokines between groups were similar. No difference was observed in edema or infiltration of inflammatory cells in pancreatic tissues from either group. However, necrosis of acinar cells was lower in the treatment group compared to the control group (3.5 *vs*. 3.75, *p*=0.015).

**CONCLUSIONS:**

Treatment with diazoxide can reduce necrosis of acinar cells in an experimental model of acute pancreatitis, but does not affect the inflammatory response or mortality after 72 h.

## INTRODUCTION

Acute pancreatitis (AP) has a broad clinical spectrum, ranging from mild symptoms to multiple-organ dysfunction syndrome and death 1,2. Regardless of cause, AP is characterized by inappropriate activation of pancreatic enzymes, necrosis of acinar cells, as well as local and systemic inflammation 2–5. Outcome is related to extension of necrosis of pancreatic tissue and the inflammatory response. In severe cases, mortality can be high (≈15%), particularly if necrotic tissue becomes infected 2,6. No specific treatment is available and, in spite of advances in intensive care and management of complications, few benefits in the outcome of this group of patients have been reported 2,7.

Diazoxide is used as an antihypertensive drug because it acts on adenosine triphosphate-sensitive potassium (KATP) channels in the membranes of cells and mitochondria 8. Nevertheless, it has also been described as possessing an anti-inflammatory effect as demonstrated in experimental and clinical research. In rats, diazoxide accelerates recovery of the mucosa in gastric ulcers 9 and reduces lesions induced by indomethacin in the small intestine 10. Also, an anti-inflammatory effect has been demonstrated if diazoxide is administered in patients undergoing coronary artery bypass grafting 11.

Therefore, considering that AP has an inflammatory component and that diazoxide has an anti-inflammatory effect, we hypothesized that diazoxide administration may modulate the inflammatory response in an experimental model of severe AP, thereby improving the outcome and recovery.

The aim of our study was to evaluate the effect of diazoxide with regard to mortality, pancreatic injury and the inflammatory response in an experimental model of severe AP.

## MATERIALS AND METHODS

The study protocol was approved by the Ethics Committee for the Use of Animals of Faculdade de Medicina da Universidade de São Paulo (protocol number 102/15).

The study group was 58 male Wistar rats (200–400 g). Animals were handled according to the *G*uidelines for the care, handling and use of laboratory animals (National Institutes of Health, Bethesda, MD, USA, 1985).

### Surgical procedures

Rats were anesthetized (ketamine hydrochloride (70 mg/kg body weight) and xylazine chloride (10 mg/1 g)) and fixed in the supine position on a platform. They underwent trichotomy and antisepsis of the abdomen using polyvinylpyrrolidone.

The tail vein was cannulated and animals divided randomly into two groups before AP induction. The control group received sterile physiologic (0.9%) saline 15 min before the surgical procedure. The treatment group received diazoxide (45 mg/kg) before the procedure.

### AP induction

After laparotomy, the duodenal arch and pancreas were externalized. Identification and occlusion of the bile duct at the level of the hepatic hilum was done using a bulldog clamp. The non-mesenteric portion of the duodenum was punctured with a needle (25 mm × 7 mm). The bile duct was catheterized with a polyethylene (PE-10) catheter. After catheterization, 0.5 mL of sodium taurocholate (2.5%) was injected in a retrograde fashion for 1 min. Then, the catheter was removed, the duodenal puncture closed (nylon 6-0), the bulldog clamp removed, and the abdominal wall closed in layers (nylon 5-0).

The type of severe AP induced by the method used in the present study is characterized macroscopically by immediate hyperemia and edema. Later, this macroscopic diagnosis was confirmed by microscopic assessment.

### Mortality curves

To evaluate mortality, animals were observed every 12 h for 72 h. Animals alive <72 h after AP were killed by anesthesia. This was followed by exsanguination by section of the abdominal aorta and inferior vena cava.

### Histology and inflammatory cytokines

One group of animals was killed 3 h after AP induction for the collection of blood samples and pancreatic tissue.

The pancreas was fixed in formalin 10% and embedded in paraffin. Tissue sections were stained by hematoxylin and eosin for histopathologic analyses under light microscopy. Schmidt’s scale was used to classify AP degree 12,13. This scale analyses edema, necrosis of acinar cells, fat necrosis, hemorrhage and inflammatory infiltration.

Expression of inflammatory cytokines in plasma were assessed using ELISA (Thermo Scientific, Waltham, MA, USA). Levels of interleukin (IL) 4, 6, 10, 13, as well as tumor necrosis factor (TNF)α were measured according to manufacturer instructions.

### Statistical analyses

Data are the mean±standard error or median (interquartile range), where appropriate. Histologic lesions were evaluated using the Mann–Whitney test. Expression of inflammatory mediators and volume of ascites were analyzed by the Student’s *t*-test or Mann–Whitney test. For mortality curves, analyses were by the log-rank test (Mantel–Cox). *p*<0.05 was considered significant.

## RESULTS

### Mortality curves

**Figure 1:**
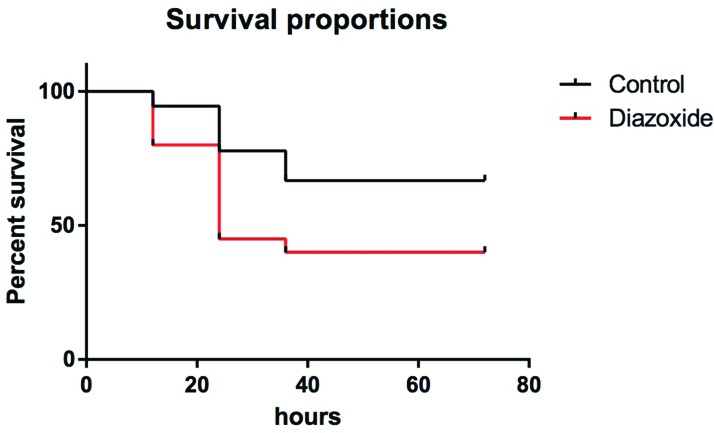
Seventy-two hours after induction of acute pancreatitis, mortality was 33% in the control group (n=18) and 60% in animals that received diazoxide (n=20) (*p*=0.076, Mantel–Cox test).

Initially, to determine the effect of diazoxide treatment on mortality, 38 rats (18 in the control group and 20 in the treatment group) underwent AP induction and were observed for ≥72 h. After this period, 6 animals (33%) in the control group had died and 12 animals (60%) in the treatment group had died (Figure 1), and the difference was not significant (*p*=0.076).

### Ascites formation and histologic analyses

There was no significant difference in the volume of ascites collected from control animals and animals treated with diazoxide (*p*=0.172).

**Figure 2:**
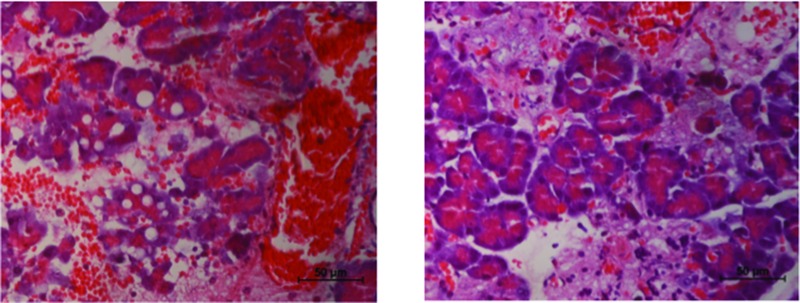
Diazoxide decreases necrosis of acinar cells in acute pancreatitis (AP). AP induction caused necrosis of multiple pancreatic acinar cells (left image). In the group that received diazoxide, there was a significant reduction in the number of necrotic cells (right image) (n=20 in each group, *p*=0.015, Mann–Whitney test).

Histologic analyses of pancreatic tissue after AP induction in the control group showed considerable interstitial edema, several hemorrhagic foci, and extensive fat necrosis (Figure 2, panel A). None of these features were modified by pretreatment with diazoxide (Figure 2, panel B; Table 1).

**Figure 3:**
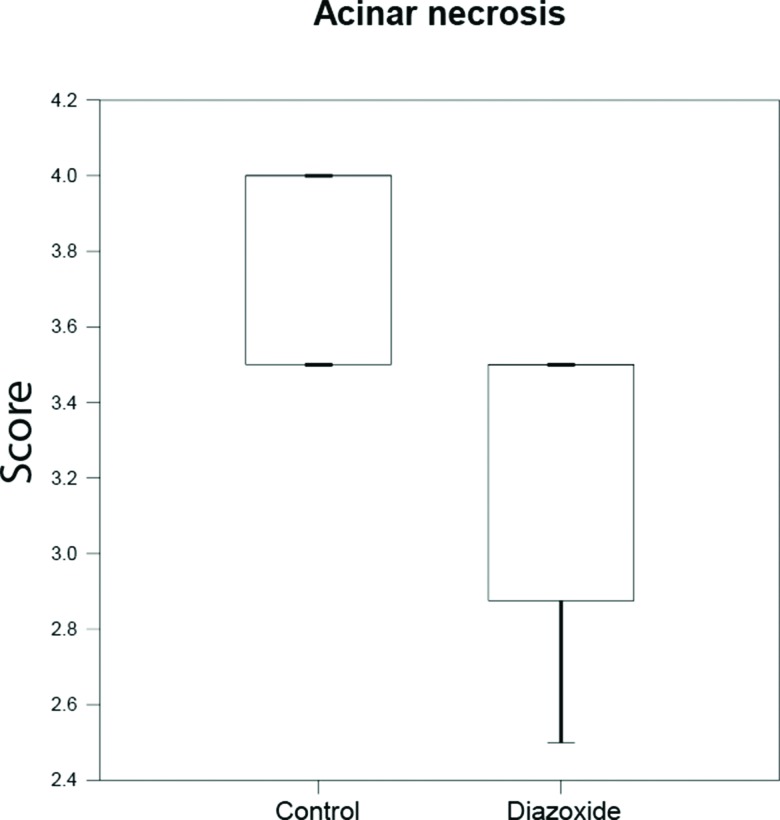
Median acinar necrosis score in control and treatment groups (diazoxide) (n=20 per group, *p*=0.015, Mann–Whitney test).

In control animals, acinar necrosis was also prominent, but it was reduced in the treatment group (Figure 2, 3).

### Inflammatory response

A significant difference was not observed in the number of inflammatory cells in pancreatic tissue after diazoxide treatment (Table 1).

**Figure 4:**
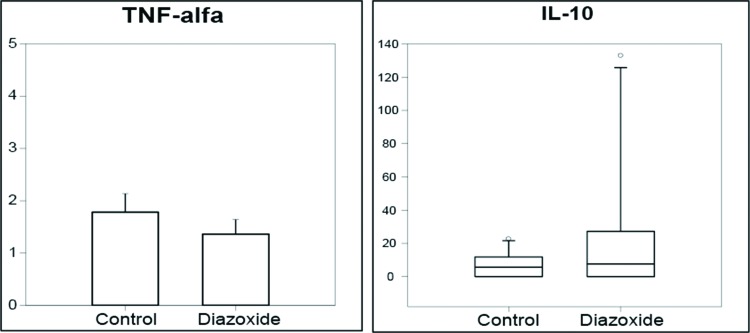
Serum concentrations of TNFα and IL10. Induction of acute pancreatitis caused an increase in serum levels of TNFα and IL-10, which was not modified by diazoxide pretreatment (n=20 in each group; *p*=0.36 and 0.44, respectively).

To assess systemic inflammation, expression of several cytokines in plasma was measured. Diazoxide treatment did not affect circulating levels of cytokines linked to the type-1 T-helper cell (Th)1 response (e.g., TNFα) nor cytokines released during a Th2 response (e.g., IL10) (Figure 4). Serum levels of other cytokines (IL1, IL4, IL13) could not be detected by ELISA in either group.

## DISCUSSION

We report that, using an experimental model of AP in Wistar rats, diazoxide can reduce necrosis of acinar cells without affecting the overall inflammatory response.

The experimental model used in the present study was effective for AP induction. Sodium taurocholate caused severe histologic damage similar to that observed for other experimental models 14–16. Significant edema, intense infiltration of leukocytes and intra-pancreatic bleeding shows the similarity of the histopathology of experimental lesions with moderate-to-severe pancreatitis in humans 14,15. Mortality in the control group at 24 h was 22%, similar to the mortality (20%) obtained using glycodeoxycholic acid (5–10 mmol) by other research teams. The different mortality obtained in the treatment group could have been related to the effects on blood pressure because this parameter was not controlled in our study.

Despite the anti-inflammatory effect of diazoxide shown in other studies, there was no effect on expression of inflammatory cytokines or pancreatic inflammatory infiltrates in the present study. This is interesting because, in other models with a prominent chronic component, such as recovery of the mucosa in gastric ulcers 9–11, diazoxide is effective whereas, in our model (which represents more acute injury), it was not.

In 20% of cases, AP presents with pancreatic and/or peri-pancreatic necrosis, the evolution of which is related to the severity and extent of pancreatic injury and presence/absence of infection 1,6,17,18. In our experimental model, diazoxide could reduce necrosis of acinar cells.

Necrotic cell death occurs generally in response to physico-chemical stress, including hypoxia, ischemia, hypoglycemia, extreme temperature changes, and nutrient deprivation 19. All these processes lead to diminished ATP production by mitochondria, increased glycolisis and intracellular acidification. The cell answers to the pH drop by activating the Na^+^/H^+^ antiporter, increasing intracellular Na^+^ levels. Since Na^+^/K^+^ ATPase is not functioning adequatly, due to lack of ATP, Na^+^ accumulates inside the cell 20. The Na^+^/Ca^2+^ channel is, then, activated, loading the intracellular millieu with Ca^2+^. Citoplasmic Ca^2+^ enters the mitochondria, which loose their capacity of controlling intracellular Ca^2+^, ensuing cell death 21.

It is known that diazoxide opens KATP channels on the plasma and mitochondrial membranes 8. This phenomenon would alleviate the initial intracellular Na^+^ accumulation, protecting the cell. Several studies have demonstrated the importance of mitochondrial KATP for protection of cells against ischemia, prevention of ATP loss, inhibition of calcium entry, and reductions in levels of reactive oxygen species 22. Other investigators have reported that opening of the mitochondrial permeability pore is involved in AP pathogenesis because it reduces ATP production and culminates in tissue necrosis 23.

Diazoxide has also been recently reported to exert antioxidant effects 24. Since redox inbalance is one of the main mechanisms responsible for the triggering of cell death, as observed in ischemia-reperfusion injury 25, we may also speculate that antioxidant action of diazoxide could be, at least partially, responsible for our findings. This hypothesis, however, was not tested in our experiments.

We can, therefore, postulate that the positive effect of diazoxide in reduction of injury to pancreatic tissue is related to its action upon the mitochondrial permeability pore.

Use of diazoxide in an experimental model of AP can reduce the intensity of necrosis of acinar cells.

## AUTHOR CONTRIBUTIONS

Andrade RO was responsible for the study design, experimental procedures and manuscript writing. Kunitake T was responsible for the study design and experimental procedures. Koike MK was responsible for the study design, experimental procedures, statistical analysis and manuscript writing. Machado MC was responsible for the study design and manuscript revision. Souza HP was responsible for the study design and manuscript revision.

## Figures and Tables

**Table 1:** Morphological features of pancreatic injury after AP induction.

Feature	Control	Treatment	*p*
Fat necrosis*	2.8±0.3	2.8±0.5	0.791
Inflammatory infiltrate*	3.6±0.1	3.4±0.2	0.247
Interstitial Edema**	4.0 (3.5-4.0)	3.5 (3.5-3.8)	0.382
Hemorrhagic foci**	4.0 (2.6-4.0)	3.5 (3.0-3.8)	0.622

* These features presented a normal distribution, therefore data are presented as Mean±SEM and the statistic test used was Student's t-test.

** These features did not present a normal distribution, therefore data are presented as Median and confidence interval, with the Mann-Whitney test being used to compare the groups.
